# *MrPIXEL*: automated execution of Pixel calculations via the *Mercury* interface

**DOI:** 10.1107/S1600576720008444

**Published:** 2020-07-30

**Authors:** Matthew G. Reeves, Peter A. Wood, Simon Parsons

**Affiliations:** a EaStCHEM School of Chemistry and Centre for Science at Extreme Conditions, The University of Edinburgh, King’s Buildings, West Mains Road, Edinburgh EH9 3FJ, Scotland; b Cambridge Crystallographic Data Centre, 12 Union Road, Cambridge CB2 1EZ, England

**Keywords:** intermolecular interactions, contact energy, lattice energy, thermodynamics

## Abstract

The interpretation of crystal structures in terms of intermolecular interaction energies enables phase stability and polymorphism to be rationalized in terms of quantitative thermodynamic models, while also providing insight into the origin of physical and chemical properties including solubility, compressibility and host–guest formation. The Pixel method is a semi-empirical procedure for the calculation of intermolecular interactions and lattice energies based only on crystal structure information. Molecules are represented as blocks of undistorted *ab initio* molecular electron and nuclear densities subdivided into small volume elements called pixels. Electrostatic, polarization, dispersion and Pauli repulsion terms are calculated between pairs of pixels and nuclei in different molecules, with the accumulated sum equating to the intermolecular interaction energy, which is broken down into physically meaningful component terms. The *MrPIXEL* procedure enables Pixel calculations to be carried out with minimal user intervention from the graphical interface of *Mercury*, which is part of the software distributed with the Cambridge Structural Database (CSD). Following initial setup of a crystallographic model, one module assigns atom types and writes necessary input files. A second module then submits the required electron-density calculation either locally or to a remote server, downloads the results, and submits the Pixel calculation itself. Full lattice energy calculations can be performed for structures with up to two molecules in the crystallographic asymmetric unit. For more complex cases, only molecule–molecule energies are calculated. The program makes use of the CSD Python API, which is also distributed with the CSD.

## Abstract 2

A program is described that enables the energetics of crystal packing to be analysed quickly and easily through a user interface, using Pixel, Python scripts and the widely known visualizer *Mercury*.

## 1. Introduction   

### 1.1. Intermolecular interactions in crystal structures   

Intermolecular interactions control an enormous diversity of chemical and physical properties in solid materials including the phase adopted under a given set of applied conditions, the solubility and melting point, and thermodynamic properties such as lattice energy, hardness, thermal expansion, heat capacity and so on. The principal aim of many crystal structure determinations, particularly in fields such as crystal engineering and polymorphism research, is to understand relative phase stability and the significance of specific intermolecular contacts including ‘structure directing’ interactions such as the carb­oxy­lic acid dimer, nitro–iodo and acid–pyridine contacts (Mukherjee, 2015 ▸). Use of these ‘synthons’ has been exploited in, for example, formation of co-crystals with active pharmaceutical ingredients with the aim of generating crystalline forms with improved performance (Aakeröy *et al.*, 2011 ▸).

Intermolecular interactions are most commonly identified and ranked by assuming that short interatomic interactions characterize stabilizing interactions, with the shortness of a contact defined relative to the sum of the van der Waals radii of the atoms in question. This method is not only quick and amenable to graphical visualization, but use of a common set of radii such as Bondi’s (1964 ▸, 1966 ▸) ‘prehistoric’ (Gavezzotti, 2013 ▸) compilation or Alvarez’s (2013 ▸) more recent and much more extensive set provides a unifying framework for discussion of intermolecular contacts.

Use of interatomic distances for interpreting crystal structures will tend to bias analyses towards those contacts in which interactions are mediated by specific atom–atom interactions. While hydrogen bonds, halogen bonds and the growing catalogue of related interactions are readily identifiable, stabilizing contacts between non- or weakly polar molecules, which are better understood in terms of whole-molecule interactions, are harder to identify. The lack of a distinctive interatomic signature in van der Waals interactions (*i.e.* those dominated by dispersion) has led to their significance being unrecognized (Dunitz & Gavezzotti, 2012*a* ▸). For example, the crystal structure of MeNSOF_2_ contains no interatomic contacts at all when judged using van der Waals radii, yet still it is a solid with intermolecular energies similar to those found for medium-strength hydrogen bonds and a lattice energy of a similar magnitude to acetic acid (−62 and −69 kJ mol^−1^, respectively; Mews & Parsons, 2014 ▸; Chickos & Acree, 2002 ▸). Focusing on prominent interactions can also give a misleading impression of the nature of an overall intermolecular contact. For example, out of 14 molecule–molecule contacts within the first coordination sphere of γ-glycine, six are destabilizing, including two which involve hydrogen bonds (Moggach *et al.*, 2015 ▸).

When considering thermodynamic stability, there are obvious advantages to working in joules rather than ångströms, and there is a growing interest in interpreting crystal structures using whole-molecule–whole-molecule energies (Dunitz & Gavezzotti, 2005 ▸; Dunitz, 2015 ▸; Mackenzie *et al.*, 2017 ▸). Quantum mechanical methods enable interaction energies to be computed to a very high level of accuracy, as illustrated by the *ab initio* calculation of the sublimation energy of benzene, but can be very time consuming for large molecules (Yang *et al.*, 2014 ▸). While most quantum mechanical methods provide a single intermolecular energy, some, including symmetry-adapted perturbation theory (Hohenstein & Sherrill, 2012 ▸; Szalewicz, 2012 ▸), break the energy down into constituent electrostatic, polarization, dispersion and Pauli repulsion terms, providing insight into the physical nature of an interaction. Though these methods are a gold standard in the field, they too are time consuming for large molecules when applied at the most accurate level.

### 1.2. The Pixel method   

The Pixel method, which was originally devised by Gavezzotti (2005 ▸, 2007 ▸, 2011 ▸), adopts a semi-empirical approach in which molecules in a crystal structure are represented by blocks of electron and nuclear density sub-divided into small cubic volume elements referred to as ‘pixels’. The molecular electron densities are calculated *ab initio* using *Gaussian* (Frisch *et al.*, 2016 ▸) at the MP2 or B3LYP level, commonly with the 6-31G** basis set using a grid of spacing 0.08 Å. To save computer time the grid is ‘condensed’ into a coarser one, typically of dimension 0.32 Å. The electrostatic energy between two molecules can then be calculated by applying Coulomb’s law to pairs of pixels from each molecule and summing the values. A similar approach can be used for the polarization, dispersion and repulsion terms to achieve a total intermolecular energy broken down into physically meaningful contributions. Application of this approach to a cluster of molecules surrounding a central reference molecule enables the lattice energy to be evaluated. The most appropriate cluster radius depends on the size and nature of the molecules but is typically between 12 and 20 Å. The accuracy of the methods has been discussed by Chickos & Gavezzotti (2019 ▸) by comparing calculated sublimation energies with a large database of the experimental values. Overall, the performance of the Pixel method is similar to that of periodic density functional theory in estimating sublimation enthalpies of organic solids, but at a fraction of the cost in terms of computing time (Maschio *et al.*, 2011 ▸).

The Pixel method has been applied to numerous systems, such as in the quantitative investigation and description of synthons for crystal engineering (Dunitz & Gavezzotti, 2012*a* ▸). Recent work using Pixel helped elucidate the role of intermolecular interactions and lattice energies for polymorphs of 5-methyl-2-[(2-nitro­phenyl)-amino]-3-thio­phene­carbo­nitrile (colloquially known as ROY) at high pressure (Funnell *et al.*, 2019 ▸). Pixel has also been used to identify and rationalize the metastable form of glycolide (Hutchison *et al.*, 2015 ▸), explain the effect of chemical substitution on halogen bonding (Carlucci & Gavezzotti, 2017 ▸), and identify the features of racemic and homochiral polymorphs that make them thermodynamically competitive (Dunitz & Gavezzotti, 2012*b* ▸).

**Figure 1:**
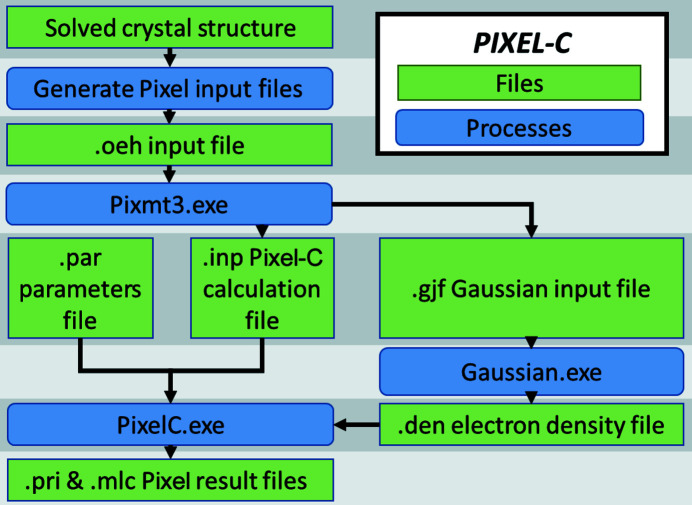
Process diagram for a Pixel calculation using the *Pixel-C* program within the *CLP-Pixel* package starting from the results of a crystal structure determination. Green boxes show files and blue boxes show processes.

This Pixel method for crystal structures is implemented in the *Pixel-C* module of the *CLP-Pixel* package (Gavezzotti, 2011 ▸), and the workflow of a Pixel calculation is shown in Fig. 1 ▸. Intermolecular energies are sensitive to H-atom positions, and if the crystal structure was determined using X-rays the distances involving hydrogen atoms should be ‘normalized’ to the more accurate typical values seen by neutron diffraction. Certain other modifications may also be necessary (see Section 2.1 below). The atomic positions, their type (*e.g.* *sp* ^2^ or *sp* ^3^ C *etc*.) and the space-group symmetry are defined in an initial setup file (.oeh). A routine (*Pixmt3*) generates both the Pixel calculation file (.inp) and the *Gaussian* job input file (.gjf) necessary to determine the electron density. *Gaussian* may be run locally or remotely to produce a cube-format electron-density file (.den). Parameters for the calculation, *e.g.* those that control the pixel size and other parameters, are stored in a separate .par file. The results of each calculation are stored in plain text .pri and .mlc files which report overall calculation results and individual dimer results, respectively.

The aim of this article is to describe a set of Python scripts that automate the process described above directly from the interface of the *Mercury* structure visualization software (Macrae *et al.*, 2020 ▸) which is distributed with the Cambridge Structural Database (CSD; Groom *et al.*, 2016 ▸). The scripts described make use of the CSD Python API. Pixel calculations are also accessible via the recently described *MiCMoS* package of computer programs, which brings together the *AA-CLP*, *CLP-Pixel* and *CLPDyn* procedures (Gavezzotti *et al.*, 2020 ▸). In addition, a procedure based on periodic electron densities is available in the program *q-GRID* (de Klerk *et al.*, 2016 ▸). Graphical visualization of Pixel results can be accomplished with the *processPIXEL* procedure (Bond, 2014 ▸).

## 2. The *MrPIXEL* process   

The program *MrPIXEL* consists of two elements. The first, *SetupPixel*, is executed from within *Mercury* and interprets the crystal structure and generates the input files required for the Pixel calculation. The second, *MrPIXEL Console*, handles all processes after initial file setup and displays the status of calculations through a graphical interface. A supplementary program, *MrPIXEL Settings*, is used to define default settings, file locations and so on.

### 2.1. Modification of the crystal structure   

Before generation of the input files, structures may require some modification in order to satisfy the requirements of the *Pixel-C* program. The number of molecules in the asymmetric unit (*Z*′) is limited to two and these must comprise complete molecules. Therefore, where a molecule occupies a special position, the space-group symmetry of the crystal structure should be reduced to a description in which the asymmetric unit consists of whole molecules. The CSD’s *Mercury* software can be used for this purpose, employing the *Change Spacegroup to Subgroup* tool found in the ‘Edit’ menu. For example, in the crystal structure of 2,2′-bi­pyridyl (CSD refcode BIPYRL; Merritt & Schroeder, 1956 ▸) the molecule occupies the inversion centre in space group *P*2_1_/*c* with an asymmetric unit that consists of half the molecule. By reducing the space group to either *Pc* or *P*2_1_ the inversion centre is removed and the asymmetric unit contains a complete molecule. It should be noted, however, that in cases where *Z*′ = 1.5 reduction of the symmetry results in *Z*′ = 3, which cannot be accommodated in a Pixel lattice energy calculation. For example, in benzidine form III (CSD refcode BENZIE02, space group *P*2_1_/*c*) one molecule occupies a general position and the other occupies an inversion centre, and no choice of reduced symmetry will satisfy the conditions for Pixel (Rafilovich & Bernstein, 2006 ▸). In cases where symmetry lowering involves an origin shift, it is advantageous to use a non-standard setting of the lower-symmetry space group to ensure that intermolecular relationships that do not involve the symmetry operations lost in the symmetry lowering are preserved exactly. There are, in short, a number of alternative strategies that may be used to lower symmetry, and for this reason no attempt has been made to implement a general procedure.

Where disorder is present, an ordered model should be constructed which contains just one component. Again, this is not handled automatically as users will normally wish to select which disorder component to keep. The required editing can be accomplished with the *Edit Structure* tool under the *Mercury* ‘Edit’ menu. Because the CSD Python API reads crystal structures upon loading into the window, the edited structure should be saved as a .cif and reloaded after any changes are made.

Once any necessary editing has been carried out, the *SetupPixel* script can be run from the *Mercury* CSD Python API menu. Upon selecting *SetupPixel*, the structure is read and interpreted, and the user is then prompted for some Pixel calculation parameters. This includes a calculation name (defaulted to the .cif title), whether to normalize hydrogen positions (required for conventionally modelled structures derived from X-ray data), and the charge and spin multiplicity of each component.

### 2.2. Generating the .oeh input file   

The user is given the option to generate the initial Pixel files only or pass the task onto *MrPIXEL Console* so that the entire calculation can be run automatically. In either case, *SetupPixel* first generates the Pixel input .oeh file. Information relating to crystal symmetry, cell parameters and atomic positions is taken from the structure as interpreted by *Mercury*. Atomic type indicators, as defined by Pixel (see Table S1 in the supporting information), use similar definitions to the Tripos .mol2 *SYBYL* typing (Tripos Inc., St Louis, MO, USA). These atomic type indicators can therefore be determined by a translation of *SYBYL* to Pixel types using a look-up table. This is particularly important for carbon atoms, for which atomic polarizabilities are assigned according to bonding environment. *SYBYL* assignments are based on the results of the CSD *auto_edit* structure tool, which adds atom- and bond-type descriptors to the structure. To store this information and to allow users to inspect the assignments, the resulting structure is saved as a .mol2 file.

It is also possible at this stage to select the level of theory and the basis set for the* Gaussian *calculations. For organic molecules containing atoms with atomic numbers up to bromine, MP2/6-31G** is usually used, while B3LYP/6-31G** is used for first-row transition-metal complexes. Different levels of theory as well as additional *Gaussian* setup settings are stored as a history that allows the user to select the correct job line as required.

### 2.3. Running *Pixmt3*   

Once written, the .oeh file is passed to the *CLP-Pixel Pixmt3* routine which generates input files for *Gaussian* and *Pixel-C*. The electron-density step size and van der Waals radius parameter values may be specified here or left as a default value set in the settings. The electron-density step size denotes the dimensions of each pixel cube calculated in *Gaussian*. The default values work well for elements up to bromine but the step size should be reduced to 0.06 Å or lower for structures involving heavier atoms (Carlucci & Gavezzotti, 2017 ▸). To reduce computational time, Pixel calculations are run using ‘super pixels’ of *n* ^3^ pixels, where *n* is known as the ‘condensation level’; *n* is usually set to 4 so that a step size of 0.08 Å produces super-pixel cubes of dimension 0.32 Å. For most purposes this condensation level is adequate but it can be changed in *MrPIXEL Settings*. Any necessary changes to the file output by *Pixmt3*, *e.g.* to the basis set, are made by *MrPIXEL Console* at this stage.

### 2.4. Generating *Gaussian* electron-density files   

*MrPIXEL Console* accommodates *Gaussian* calculations performed both locally and on remote cluster installations. For remote jobs, *MrPIXEL Console* interfaces with clusters through the Python SSH module *Paramiko. Paramiko* enables the scripts to connect securely to remote clusters via an SSH key combination. The username and password are only needed for initial setup of the key files on both the local and remote locations. Future connections match these key files. The details of submission and retrieval depend on local cluster type and administration policy. The scripts used in Edinburgh are included in the *MrPIXEL* package, but we expect that these will usually need some modification in other locations.

The cluster address and folder locations can be specified in *MrPIXEL Settings*. In the system implemented in Edinburgh, *Gaussian* jobs are submitted using a Bash script, and a template Bash file should be included in the PIXEL\Batch\ folder on the local system. *MrPIXEL Console* produces an edited copy of this for each calculation, replacing the entry Name.gjf with the job file name. Calculations are checked periodically when running *MrPIXEL Console* and the density files are downloaded when *Gaussian* jobs are complete. A job is deemed complete when the required electron-density ‘cube’ file is retrieved.

### 2.5. Running the Pixel calculation   

Following retrieval of the density file, the Pixel calculation is called by *MrPIXEL*. Pixel calculations are carried out using the *Pixel-C* module of the *CLP-Pixel* suite, using all available CPU resources on a single core. It is not recommended to run calculations concurrently that equal or exceed the core count on a user’s machine. The user may therefore specify a maximum number of cores available to *Pixel-C* tasks in the settings menu, which will result in *MrPIXEL* queueing tasks that exceed this limit until a free core is available. *MrPIXEL Console* reports the completion of *Pixel-C* tasks in the graphical user interface and provides functionality to view the interactions as sorted tables in a text viewer under the *MrPIXEL Console* ‘Structure’ menu.

## 3. Examples   

### 3.1. The first coordination sphere of γ-glycine   

**Figure 2:**
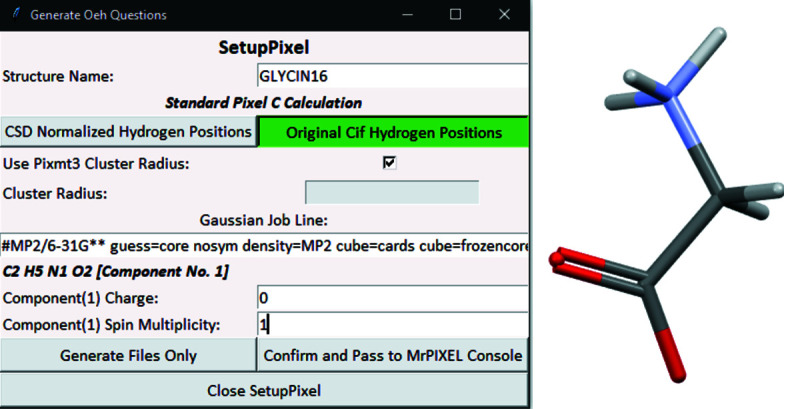
A *SetupPixel* window for the GLYCIN16 structure showing typical settings.

A straightforward calculation may be demonstrated using the structure of γ-glycine determined by neutron diffraction (CSD refcode GLYCIN16; Kvick *et al.*, 1980 ▸). The structure contains one molecule in the asymmetric unit which occupies a general position in the space group *P*3_2_. No modification to the space group is necessary, and *SetupPixel* can be called without any manual modification (Fig. 2 ▸).

The *SetupPixel* menu defines settings for the Pixel calculation. The first line describes the job title and may be changed as required; the default is taken from the structure data title in the CIF. For CSD entries this comes from the CSD refcode. The next line defines the job type. If *Z*′ ≤ 2 this will be a standard Pixel calculation. Where more than two mol­ecules occupy the asymmetric unit, a series of separate Pixel calculations are carried out with pairs of components (see below). The user then selects whether to normalize hydrogen positions (as is typical for X-ray data). In this example, GLYCIN16 was determined using neutron data and so normalization is not needed. The next option determines whether the cluster radius should be as determined by *Pixmt3* or defined by the user. For the present calculation, our interest is only in the dimer energies of the first coordination sphere, and the *Pixmt3* cut-off (14 Å) is more than adequate. Finally, the charges and spin-multiplicity values are required for each molecule. In this case, the defaults (charge = 0, spin multiplicity = 1) are correct for the glycine molecule.

The calculation is passed to *MrPIXEL*. The user is prompted for the electron-density grid size for the cube-format file generated in the *Gaussian* calculation. A *Gaussian* job is then submitted and its progress is monitored; when complete, the cube file is downloaded and the Pixel calculation is initiated.

The calculation returns a lattice energy of −235.9 kJ mol^−1^, though it should be noted that glycine is zwitterionic in the solid state and contact energies at the cut-off radius of 14 Å still have interaction energies in excess of 2 kJ mol^−1^ as a result of long-range electrostatic interactions. The experimental lattice energy of glycine is between −136 and −139 kJ mol^−1^, the large difference with the calculated value reflecting the transfer of a proton between the ammonium and carboxyl­ate groups which occurs in the gas phase (Chickos & Acree, 2002 ▸). The lattice energy of glycine neglecting the proton transfer has been estimated to be −290 (8) kJ mol^−1^ (Raabe, 1999 ▸).

Molecules in the first coordination sphere can be identified from nonzero values of the repulsion or dispersion energies, which are very short range interaction terms. There are 14 molecules in the first coordination sphere (Table 1 ▸), the strongest interaction being the head-to-tail hydrogen bond formed between the ammonium and carboxyl­ate groups which forms a C(5) chain along the *c* axis. The interaction is dominated by the electrostatic term (−119.2 kJ mol^−1^), with a smaller contribution from dispersion, as is typical for hydrogen bonds. The pattern of interactions can also be visualized with the *ProcessPIXEL* software (Bond, 2014 ▸; Shishkin *et al.*, 2012 ▸) (see the supporting information for details).

### 3.2. The lattice energy of ethyl­ene   

The space group of the crystal structure of ethyl­ene (CSD refcode ETHLEN10) is *P*2_1_/*n* with the molecule located on an inversion centre (*Z*′ = 1/2) (Nes & Vos, 1979 ▸). The space-group symmetry needs to be reduced to a *Z*′ = 1 description, either in *P*2_1_ or *P* *c*. This step should be carried out before setting up the Pixel calculation and can be accomplished in *Mercury* as described above (Section 2.1). The updated structure should be saved as a CIF which can be opened within the same *Mercury* window before running *SetupPixel*.

**Figure 3:**
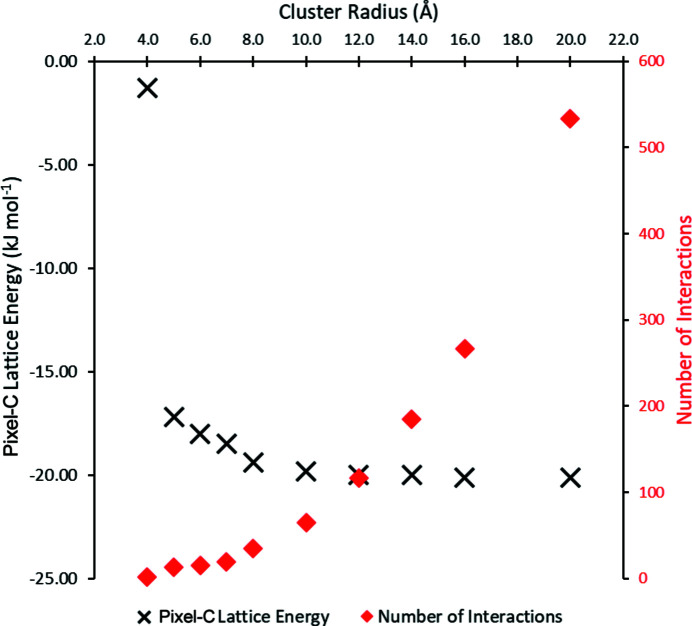
The influence of cluster radius on lattice energy for the ETHLEN10 structure as calculated by Pixel (black). The red points show the number of interactions considered at each choice of cluster radius.

The structure was determined using X-rays, and so hydrogen atoms should be normalized. The influence of the cut-off radius on the lattice energy and number of interactions is shown for ethyl­ene in Fig. 3 ▸. The lattice energy hardly changes beyond 10 Å because the influence of electrostatic contributions is very low. In practice, validation of the cut-off can be carried out after Pixel calculation is complete by checking that interaction energies at the longest distances are zero (Table 2 ▸).

The calculated lattice energy is −22.4 kJ mol^−1^, comparable to the experimental values of between −20.2 and −25.2 kJ mol^−1^. The breakdown of the contributions to the lattice energy is (in kJ mol^−1^) −5.8 for the electrostatic energy, −1.5 for polarization, −27.6 for dispersion and 12.5 for repulsion. The first coordination sphere contains 12 contacts, the interactions having a fairly uniform distribution of energies.

### 3.3. A transition-metal complex   

**Figure 4:**
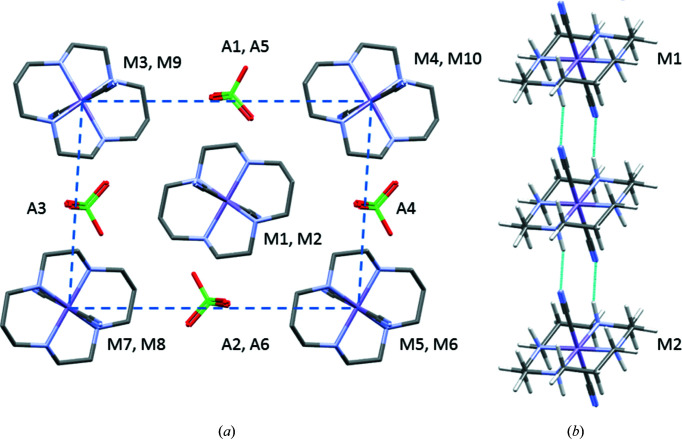
(*a*) The first coordination sphere of the cations in the Mn complex AFAROO structure. The dashed line shows the top face of the distorted cube referred to in the text. (*b*) Hydrogen bonding forming chains of cations.

Parameterization and application of the Pixel method to transition-metal complexes has been carried out using electron densities calculated via B3LYP/6-31G** calculations (Maloney *et al.*, 2015 ▸, 2016 ▸), and this is specified when running *SetupPixel*, as shown in Fig. S1(iii) in the supporting information. For the low-spin Mn(+3)-containing salt [Mn(cyclam)(CN)_2_]ClO_4_ (CSD refcode AFAROO) the spin multiplicity is 3 for the cation, while the charges on the cation and anion are +1 and −1 (Mossin *et al.*, 2002 ▸). The energies from Pixel are classified according to whether they are cation–cation, cation–anion, anion–cation or anion–anion interactions. The composition of the first coordination sphere of the cation is shown in Table 3 ▸ and in Fig. 4 ▸(*a*), where the central cation, labelled M, makes contacts to ten other cations and six perchlorate anions, labelled A1, A2 *etc*. Cations M3 to M8 are distributed in a distorted cube about the central cation. The interactions are dominated by dispersion with total energies in the range −9.1 to −19.1 kJ mol^−1^. Two pairs of anions lie at the edges of the cube, with the remaining two anions (A3 and A4) occupying the opposite faces. Topologically, it is similar to the CoO structure (Tombs & Rooksby, 1950 ▸). The strongest contacts (−66.5 kJ mol^−1^) are formed to two cations (M1 and M2) located in the top and bottom faces of the cube, connected by pairs of NH⋯NC hydrogen bonds between the cyclam and cyano ligands [Fig. 4 ▸(*b*)]. Unusually for an ionic material, electrostatics appear to play a minor role, while the most strongly stabilizing electrostatic interaction (to M1 and M2) is formed between two cations. These results reflect the distribution of a single positive charge over a relatively large cation and the retention of a significant negative electrostatic potential in the region of the cyano ligand, which Mulliken analysis shows to carry a charge of approximately −0.5*e*.

### 3.4. Pixel calculations when *Z*′ > 2   

For structures with more than two molecules in the asymmetric unit, a standard Pixel calculation is not possible (see Section 2.1). It is, however, possible to run multiple Pixel calculations to obtain individual dimer energies in the structures by consideration of substructures consisting of all possible pairs of molecules in the asymmetric unit. *SetupPixel* will recognize such structures, as shown in Fig. S1(iv), notify the user and run iterations of Pixel to generate all the dimer energies out to the set cut-off range. The setup of calculations is the same as usual but the output folder will contain calculation files for each possible combination of molecules. Note that the lattice energies obtained in these calculations are meaningless and so only relatively short cut-off radii are required.

**Figure 5:**
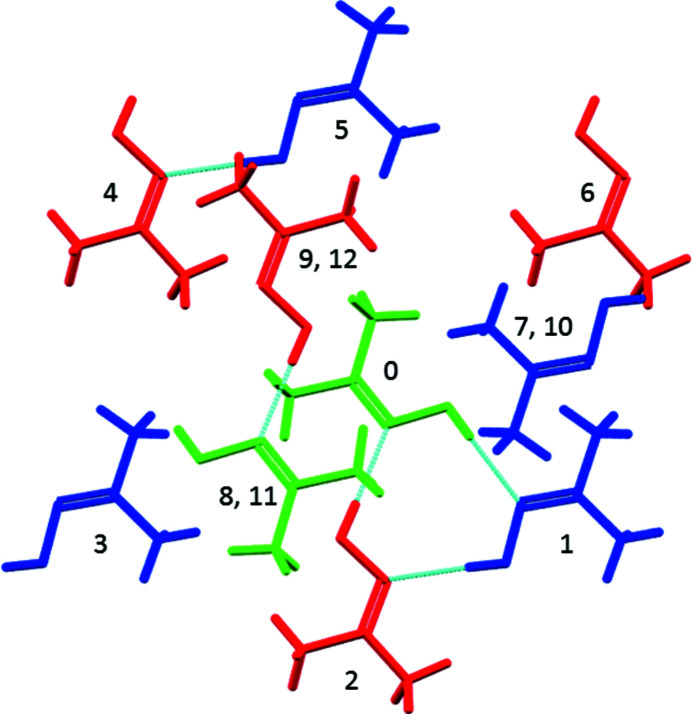
The first coordination sphere of molecule a in acetoxime shown in green, with molecules b and c shown in blue and red. The central reference molecule is labelled 0, with other contacts being labelled in the same order as Table 4 ▸. Molecules 7 and 10, 9 and 12, and 8 and 11 superimpose in this projection along **a**.

This process can be applied to the structure of acetoxime (CSD refcode ACEOXM01, Me_2_C=NOH; Parsons *et al.*, 2004 ▸), which has three molecules in the asymmetric unit in space group 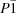. The output for this example contains three *Pixel-C* calculation results (corresponding to interactions between molecules labelled a and b, a and c, and b and c). The structure, which at 220 K has unit-cell dimensions *a* = 7.01, *b* = 10.48, *c* = 10.58 Å, α = 60.5, β = 79.6, γ = 83.5°, appears to be a distorted version of a hexagonal room-temperature phase which forms in *P*6_3_/*m* with dimensions *a* = 10.61, *c* = 7.02 Å (Bierlein & Lingafelter, 1951 ▸). The first coordination sphere of each of the three molecules in the asymmetric unit contains 12 molecules, consisting of a central layer in which each molecule is surrounded by six others generated by lattice translations. Layers above and below are related to the central molecule by inversion operations and to each other by lattice translations along **a** (Fig. 5 ▸). Overall, the arrangements have the characteristic ABAB… layer stacking of hexagonal close packing. The hexagonal close packed (h.c.p.) topology allows equivalent contacts to be identified and compared (Table 4 ▸). The h.c.p. arrangement is distorted in the parent phase by the non-spherical geometry of the molecules and hydrogen bonding between the members of the asymmetric unit, but each of the contacts in the horizontal rows in Table 4 ▸ would have been equivalent in the parent phase and show still further variation. The hydrogen bonds in the first two rows of the table are dominated by the electrostatic contribution and show less variation than the interactions between the layers (the bottom six rows) which are dominated by dispersion, illustrating the flexible character of dispersion interactions. The sums of the contacts in the three *E*_TOT_ columns are −124.8, −125.3 and −125.5 kJ mol^−1^, demonstrating the mutual compensation of the distortions that occur about each molecule.

## 4. Conclusions and program availability   

The availability of accurate semi-empirical methods such as Pixel and *CrystalExplorer* (Mackenzie *et al.*, 2017 ▸) for the calculation of intermolecular interaction energies in crystal structures provides thermodynamic insight into the intermolecular interactions which drive and determine crystal structure formation. They can be used to help interpret individual crystal structures, to compare the structures of different polymorphs, cocrystals and solvates, and to quantify the effect of chemical substitution on interactions in a series of related materials. They are broadly applicable to a range of different compounds, rather than being limited to certain classes as are some molecular mechanics methods. Compared with fully *ab initio* quantum mechanical methods, not only are they extremely fast but they also provide a breakdown of intermolecular energies into chemically meaningful terms.

Long-standing methods for understanding crystal structures, such as the use of van der Waals radii to identify stabilizing contacts, provide a way to identify atom–atom contacts and instantly place their distances in the context of similar interactions (Thakur *et al.*, 2015 ▸). While the speed of such calculations is likely to ensure they will remain the first step of most crystal structure analyses, the calculation of energies is highly complementary and enriches the information content of a crystal structure. By emphasizing molecule–molecule over atom–atom interactions it also simplifies the analysis of crystal structures by reducing the volume of numerical data that need to be considered.

The aim of *MrPIXEL* is to facilitate the development of a purely structural view of intermolecular interactions and crystal packing into a more fundamental thermodynamic view. Once the program is installed and set up, the entire process of a Pixel calculation can be carried out with the minimum of effort from the interface of *Mercury*. The code is open source and freely available from http://www.crystal.chem.ed.ac.uk/software/mrpixel. The package includes the programs *Pixel-C* and *Pixmt3* from *CLP-Pixel*, the full version of which can now be downloaded from http://www.crystal.chem.ed.ac.uk.

## Supplementary Material

Supporting information. DOI: 10.1107/S1600576720008444/kc5113sup1.pdf

## Figures and Tables

**Table 1:** A breakdown of *Pixel-C* results for the first coordination sphere of γ-glycine (GLYCIN16) Note that the small discrepancy (0.1 kJ mol^−1^ difference) in the pair of interactions at a distance of 4.389 Å is caused by rounding errors.

Symmetry operation	Centroid–centroid distance (Å)	*E*_ELEC_ (kJ mol^−1^)	*E*_POL_ (kJ mol^−1^)	*E*_DISP_ (kJ mol^−1^)	*E*_REP_ (kJ mol^−1^)	*E*_TOT_ (kJ mol^−1^)
*x*, *y*, *z* − 1	5.473	−119.2	−38.6	−14.3	66.3	−105.8
*x*, *y*, *z* + 1	5.473	−119.2	−38.6	−14.3	66.3	−105.8
−*x* + *y*, −*x* − 1, *z* + 2/3	5.458	−37.5	−11.2	−8.1	9.5	−47.5
−*y* + 1, *x* − *y*, *z* − 2/3	5.458	−37.5	−11.2	−8.1	9.5	−47.5
−*x* + *y*, −*x* − 1, *z* − 1/3	4.450	49.7	−8.7	−10.5	7.1	37.7
−*y* + 1, *x* − *y*, *z* + 1/3	4.450	49.7	−8.7	−10.5	7.1	37.7
−*y* + 1, *x* − *y*, *z* − 1/3	4.389	−28.3	−41.0	−18.5	54.4	−33.4
−*x* + *y* + 1, −*x* + 1, *z* + 1/3	4.389	−28.3	−41.0	−18.5	54.4	−33.3
−*y*, *x* − *y*,* z* + 2/3	5.437	−23.6	−11.0	−7.2	10.2	−31.6
−*y*, *x* − *y*, *z* − 2/3	5.437	−23.6	−11.0	−7.2	10.2	−31.6
−*x* + *y*, −*x*, *z* + 1/3	4.424	29.7	−33.7	−17.1	33.3	12.2
−*y*, *x* − *y*,* z* − 1/3	4.424	29.7	−33.7	−17.1	33.3	12.2
−*y* + 1, *x* − *y*, *z* + 2/3	5.408	11.0	−4.4	−3.6	1.0	4.0
−*y* + 1, *x* − *y* + 1, *z* − 2/3	5.408	11.0	−4.4	−3.6	1.0	4.0

**Table 2:** A breakdown of the *Pixel-C* results for the first coordination sphere of ethyl­ene (ETHLEN10)

Symmetry operation	Centroid–centroid distance (Å)	*E*_ELEC_ (kJ mol^−1^)	*E*_POL_ (kJ mol^−1^)	*E*_DISP_ (kJ mol^−1^)	*E*_REP_ (kJ mol^−1^)	*E*_TOT_ (kJ mol^−1^)
*x*, *y*, *z* − 1	4.067	−1.4	−0.4	−5.8	3.0	−4.7
*x*, *y*, *z* + 1	4.067	−1.4	−0.4	−5.8	3.0	−4.7
−*x* + 1/2, *y* − 1/2, −*z* + 1/2	4.441	−0.4	−0.2	−4.7	2.1	−3.2
−*x* + 1/2, *y* + 1/2, −*z* + 1/2	4.441	−0.4	−0.2	−4.7	2.1	−3.2
−*x* − 1/2, *y* − 1/2, −*z* − 1/2	4.441	−0.4	−0.2	−4.8	2.1	−3.3
−*x* − 1/2, *y* + 1/2, −*z* − 1/2	4.441	−0.4	−0.2	−4.8	2.1	−3.3
−*x* − 1/2, *y* − 1/2, −*z* + 1/2	4.600	−1.4	−0.3	−2.9	1.7	−3.0
−*x* − 1/2, *y* + 1/2, −*z* + 1/2	4.600	−1.4	−0.3	−2.9	1.7	−3.0
−*x* + 1/2, *y* − 1/2, −*z* − 1/2	4.600	−1.5	−0.3	−2.9	1.7	−3.1
−*x* + 1/2, *y* + 1/2, −*z* − 1/2	4.600	−1.5	−0.3	−2.9	1.7	−3.1
*x* − 1, *y*,* z*	4.626	−0.7	−0.2	−3.5	1.8	−2.6
*x* + 1, *y*, *z*	4.626	−0.7	−0.2	−3.5	1.8	−2.6

**Table 3:** A breakdown of the *Pixel-C* results for the first coordination sphere of the cations in AFAROO M = the central reference cation, while M1, M2… are cations related by the operations listed. A1, A2… are anions.

Contact (see Fig. 4 ▸)	Symmetry operation	Centroid–centroid distance (Å)	*E*_ELEC_ (kJ mol^−1^)	*E*_POL_ (kJ mol^−1^)	*E*_DISP_ (kJ mol^−1^)	*E*_REP_ (kJ mol^−1^)	*E*_TOT_ (kJ mol^−1^)
M⋯M1	*x* − 1, *y*, *z*	6.760	−60.6	−33.2	−36.7	64.0	−66.5
M⋯M2	*x* + 1, *y*, *z*	6.760	−60.6	−33.2	−36.7	64.0	−66.5
M⋯M3	−*x*, *y* − 1/2, −*z* + 3/2	8.668	−8.1	−4.8	−15.0	8.8	−19.1
M⋯M4	−*x*, *y* + 1/2, −*z* + 3/2	8.668	−8.1	−4.8	−15.0	8.8	−19.1
M⋯M5	*x* − 1/2, −*y* + 1/2, −*z* + 2	8.502	0.2	−4.2	−13.8	5.4	−12.4
M⋯M6	*x* + 1/2, −*y* + 1/2, −*z* + 2	8.502	0.2	−4.2	−13.8	5.4	−12.4
M⋯M7	*x* − 1/2, −*y* − 1/2, −*z* + 2	9.442	−1.6	−1.1	−10.5	3.8	−9.4
M⋯M8	*x* + 1/2, −*y* − 1/2, −*z* + 2	9.442	−1.6	−1.1	−10.5	3.8	−9.4
M⋯M9	−*x* + 1, *y* − 1/2, −*z* + 3/2	8.900	−0.0	−2.3	−15.8	9.0	−9.1
M⋯M10	−*x* + 1, *y* + 1/2, −*z* + 3/2	8.900	−0.0	−2.3	−15.8	9.0	−9.1
M⋯A1	*x*, *y*, *z*	5.359	−14.7	−8.2	−26.5	33.0	−16.3
M⋯A2	−*x* + 1/2, −*y*, *z* + 1/2	5.371	−11.5	−6.9	−27.1	29.7	−15.8
M⋯A3	−*x*, *y* − 1/2, −*z* + 3/2	6.546	−0.6	−1.8	−14.8	7.6	−9.7
M⋯A4	−*x*, *y* + 1/2, −*z* + 3/2	6.928	−0.0	−1.3	−13.2	6.5	−8.0
M⋯A5	*x* + 1, *y*, *z*	6.632	−1.1	−0.8	−10.2	3.5	−8.5
M⋯A6	−*x* − 1/2, − *y*, *z* + 1/2	6.327	−2.7	−1.7	−14.9	9.4	−9.9

**Table 4:** Comparison of the strongest interaction energies in the crystal structure of acetoxime The energies are in kJ mol^−1^.

Molecule a			Molecule b			Molecule c		
Dimer	Distance (Å)	*E*_TOT_	Dimer	Distance (Å)	*E*_TOT_	Dimer	Distance (Å)	*E*_TOT_
b	5.935	−28.1	c	5.942	−27.6	a	5.964	−28.3
c	5.964	−28.3	a	5.935	−28.1	b	5.942	−27.6
b[*x*, *y* − 1, *z*]	6.077	−4.4	c[*x*, *y* + 1, *z* − 1]	6.187	−4.1	a[*x*, *y*, *z* + 1]	6.102	−4.3
c[*x*, *y*, *z* − 1]	6.102	−4.3	a[*x*, *y* + 1, *z*]	6.077	−4.4	b[*x*, *y* − 1, *z* + 1]	6.187	−4.1
b[*x*, *y*, *z* − 1]	6.274	−3.7	c[*x*, *y* + 1, *z*]	6.170	−4.5	a[*x*, *y* − 1, *z* + 1]	6.238	−4.0
c[*x*, *y* + 1, *z* − 1]	6.238	−4.0	a[*x*, *y*, *z* + 1]	6.274	−3.7	b[*x*, *y* − 1, *z*]	6.170	−4.5
b[−*x* − 1, −*y*, −*z*]	5.033	−7.4	c[−*x* − 1, −*y* − 1, −*z* + 1]	4.631	−10.8	a[−*x* − 1, −*y* − 1, −*z*]	5.354	−6.7
a[−*x* − 1, −*y* − 1, −*z*]	4.649	−10.3	b[−*x* − 1, −*y*, −*z*]	5.356	−7.5	c[−*x* − 1, −*y* − 1, −*z* + 1]	4.540	−10.6
c[−*x* − 1, −*y* − 1, −*z*]	5.354	−6.7	a[−*x* − 1, −*y*, −*z*]	5.033	−7.3	b[−*x* − 1, −*y* − 1, −*z* + 1]	4.631	−10.8
b[−*x*, −*y*, −*z*]	5.394	−7.1	c[−*x*, −*y* − 1, −*z* + 1]	5.137	−7.4	a[−*x*, −*y* − 1, −*z*]	4.565	−11.3
a[−*x*, −*y* − 1, −*z*]	4.754	−9.2	b[−*x*, −*y*, −*z*]	4.299	−12.8	c[−*x*, −*y* − 1, −*z* + 1]	5.543	−5.9
c[−*x*, −*y* − 1, −*z*]	4.565	−11.3	a[−*x*, −*y*, −*z*]	5.394	−7.1	b[−*x*, −*y* − 1, −*z* + 1]	5.137	−7.4
