# Supplementary material for: MrPIXEL: automated execution of Pixel calculations via the Mercury interface
Source: J Appl Crystallogr. 2020 Jul 30;53(Pt 4):1154–62. doi: 10.1107/S1600576720008444 (PMC7401789; doi:10.1107/S1600576720008444)
Supplement: Supplementary file 1 [file j-53-01154-sup1.pdf]

JOURNAL OF
APPLIED
CRYSTALLOGRAPHY

Volume 53 (2020)

Supporting information for article:

MrPIXEL: automated execution of Pixel calculations via the Mercury Interface

Matthew G. Reeves, Peter A. Wood and Simon Parsons

SUPPLEMENTARY INFORMATION

MrPIXEL: Automated Execution of Pixel Calculations via the Mercury Interface

Matthew G. Reeves,[1] Peter A. Wood*[2] and Simon Parsons*[1]

1. Centre for Science at Extreme Conditions and EaStCHEM School of Chemistry, The University of Edinburgh, King's Buildings, West Mains Road, Edinburgh, Scotland, EH9 3FD, UK.

2. Cambridge Crystallographic Data Centre, 12 Union Road, Cambridge, England, CB2 1EZ, UK.

Contents

Table S1: Pixel identifiers for different atom types. Mol2 format SYBYL notations are included, as well as some custom labels denoted by * assigned by SetupPixel. This table is based on that in the Pixel manual.

Figure S1: Example menus of SetupPixel and MrPIXEL. (i). SetupPIXEL for GLYCIN16 structure, (ii) symmetry lowering for ethylene, (iii) a two-component system where the cation is a transition metal complex, and (iv) a system for which *Z*' = 3.

Notes: Visualisation of Results with ProcessPixel

Table S1: Pixel identifiers for different atom types. Mol2 format SYBYL notations are included, as well as some custom labels denoted by * assigned by SetupPixel. This table is based on that in the Pixel manual.

**Atom descriptor**	***.mol2*** **SYBYL notation**	***.oeh*** **indicator**	**Atomic Polarizability (Å³)**	**Ionization Potential (a.u.)**	**Space diffusion Parameter**	**H-bond Propensity**
**hydrogen radius 1.10**			**0.39**	**0.5**		
acetylene CH	H.1	1			0.6	0.2
$=CH_2$, arom.CH	H.2	2			0.62	0.1
aliphatic CH, CH_2, CH_3	H.3	3			0.64	0.05
R-OH, R-SH alcohol, thiol	H.5	5			0.75	0.99
COO-H acid	H.6	6			0.8	0.99
CON)-H amide	H.am	7			0.8	0.9
R_2NH, RNH_2, $(R_3N+)H$	H.8	8			0.8	0.99
H2O (water)	H2.o	9			0.8	0.99
unnormalized hydrogen atom from Cambridge files		99				
**carbon 1.77**				**0.414**	**1**	**0**
carbonyl C=(O)	C.O	10	1.05			
≡C-	C.1	11	1.35			
sp^2 or allene C	C.2, C.ar	12	1.35			
sp^3 C	C.3	13	1.05			
aromatic core C	C.Core	14	1.9			
**nitrogen 1.64**			**0.95**	**0.534**		
$(RnH_4\text{-}n)N+$	N.4	16			0.63	0
$(RnH_3\text{-}n)N$	N.3	17			0.63	-0.97
arom.N, R=N(H)	N.ar,N.pl3	18			0.58	-0.99
-C≡N,-N=N	N.2	19			0.7	-0.7
nitro N	N.1	20			0.63	0
amide N (CONH,$CONH_2$)	N.am	21			0.63	-0.85
**oxygen 1.58**			**0.75**	**0.5**		
-O-	O.3	23			0.45	-0.9
H_2O (water)	O.1	24			0.7	-0.99
C=O, COO-	O.co2, O.4,O.2	27			0.5	-0.99
(C=O)-OH	O.car	28			0.5	-0.9
R-OH	O.al	29			0.45	-0.99
N=O	O.2	30			0.5	-0.95
S=O	O.2	31			0.75	-0.9
P=O	O.3	32			0.75	-0.9
**sulfur 1.81**			**3**	**0.381**		
-S-	S.3	34			2	-0.5
(C)=S	S.1	35			2	-0.5
(O)=S	S, S.o2	36			2.5	0
R-S(H)	S.2	37			2	-0.5
**heteroatoms**						
P 1.9	P	38	1.54	0.386	3	0
As 1.8	As	39	3.5	0.4	5	0
Se 1.8	Se	40	3.5	0.4	6	0
F 1.46	F.0	41	0.55	0.64	0.2	0
Cl 1.76	Cl.0	42	2.5	0.477	2.4	-0.2

Table S1 Continued							
**atom_descriptor**		***.mol2* SYBYL notation**	**indicator**	**atomic**	**ionization**	**Space diffusion**	**H-bond**
Br	1.87	Br.0	43	3.27	0.434	1.5	0
I	2.03	I.0	44	5	0.384	5	0
B	1.70	B	46	1.6	0.305	1	0
Si	2.00	Si	47	2	0.28	1	0
**transition metals**							
Ti		Ti	51	4.18	0.25	0.8	-0.5
V		V	52	3.31	0.25		
Cr		Cr, Cr.oh, Cr.th	53	2.86	0.25		
Mn		Mn	54	2.93	0.27		
Fe		Fe	55	2.81	0.29		
Co		Co, Co.oh	56	2.62	0.29		
Ni		Ni	57	2.61	0.28		
Cu		Cu	58	2.81	0.285		
Zn		Zn	59	3.63	0.345		
**positive ions**							
Li+		Li	61	0.1	1	0.2	0
Na+		Na	62	0.2	0.85	0.3	
K+		K	63	0.3	0.7	1.5	
Rb+		Rb	64	0.4	0.5	3	
Cs+		Cs	65	0.3	0.45	5	
Ca+		Ca	66	0.7	0.7	1.5	
**negative ions**							
F-		F.-1	67	0.4	0.75	0.5	
Cl-		Cl.-1	68	2.5	0.65	3	
Br-		Br.-1	69	3.27	0.5	4	
I-		I.-1	70	5	0.4	5	

i.

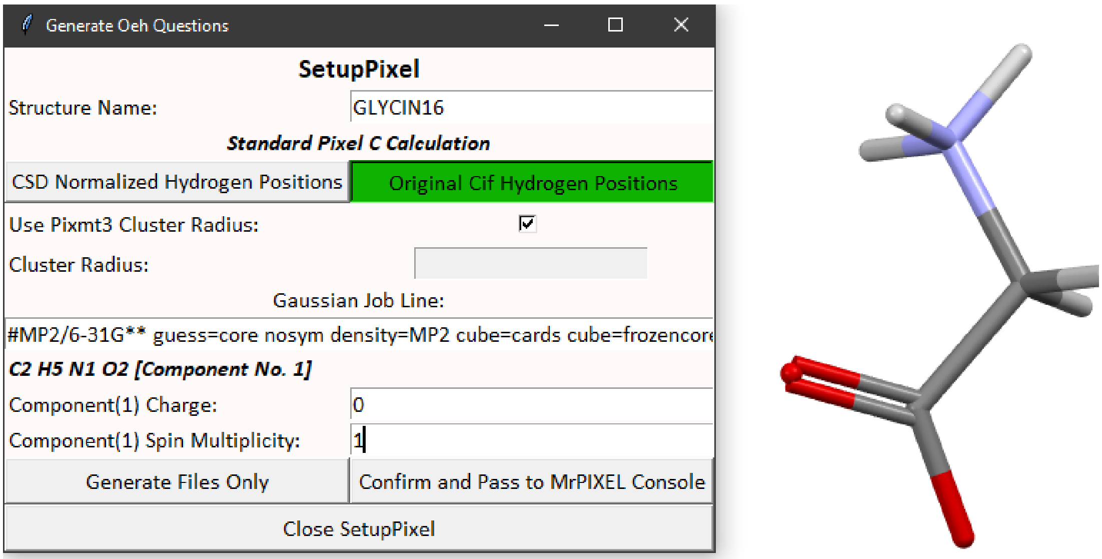
Generate Oeh Questions
SetupPixel
Structure Name:
GLYCIN16
Standard Pixel C Calculation
CSD Normalized Hydrogen Positions
Original Cif Hydrogen Positions
Use Pixmt3 Cluster Radius:
Cluster Radius:
Gaussian Job Line:
#MP2/6-31G** guess=core nosym density=MP2 cube=cards cube=frozencore
C2 H5 N1 O2 [Component No. 1]
Component(1) Charge:
0
Component(1) Spin Multiplicity:
1
Generate Files Only
Confirm and Pass to MrPIXEL Console
Close SetupPixel

ii.

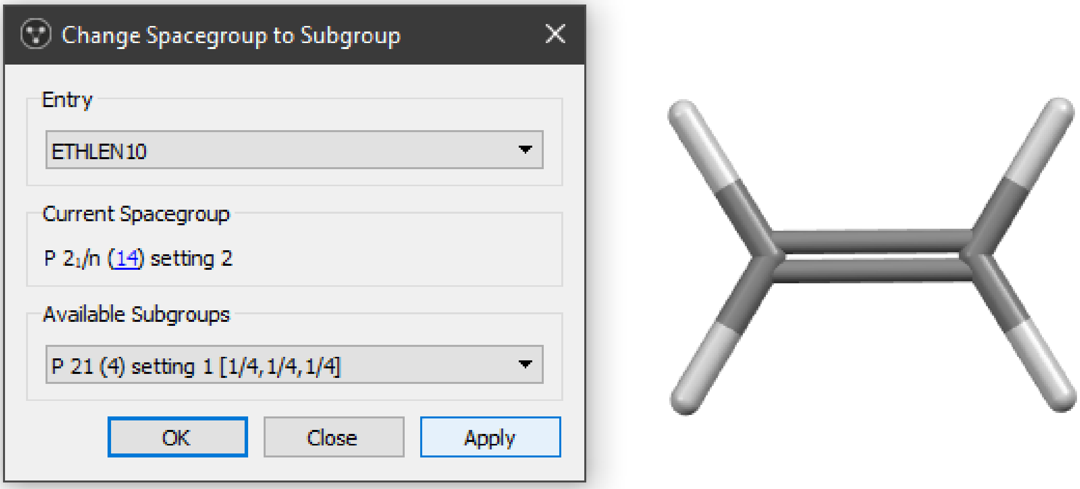
Change Spacegroup to Subgroup
Entry
ETHLEN10
Current Spacegroup
P 2₁/n (14) setting 2
Available Subgroups
P 21 (4) setting 1 [1/4, 1/4, 1/4]
OK
Close
Apply

iii.

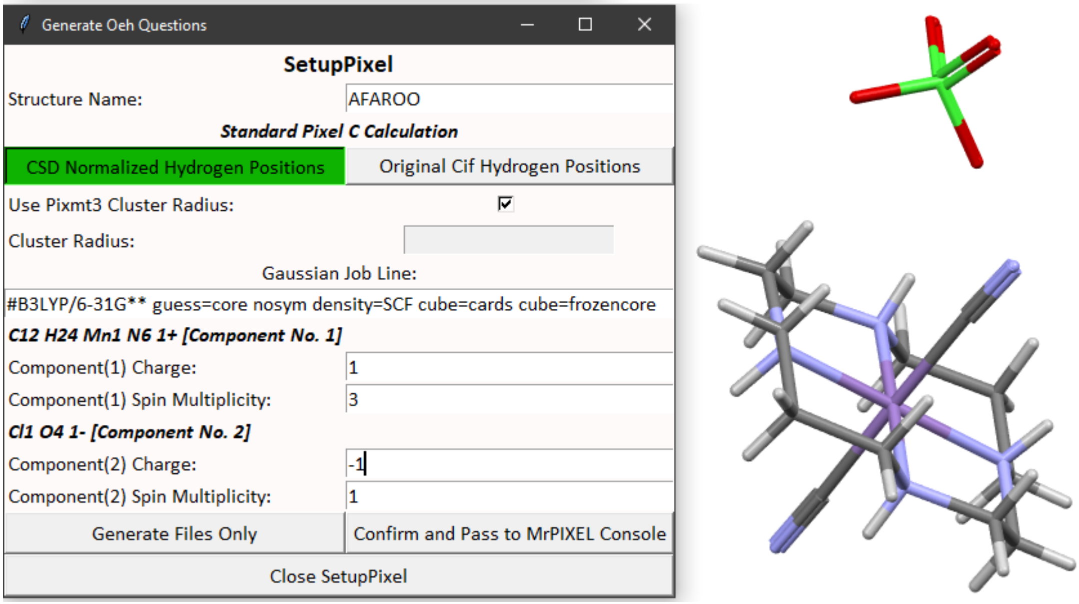
Generate Oeh Questions
SetupPixel
Structure Name:
AFAROO
Standard Pixel C Calculation
CSD Normalized Hydrogen Positions
Original Cif Hydrogen Positions
Use Pixmt3 Cluster Radius:
Cluster Radius:
Gaussian Job Line:
#B3LYP/6-31G** guess=core nosym density=SCF cube=cards cube=frozencore
C12 H24 Mn1 N6 1+ [Component No. 1]
Component(1) Charge:
1
Component(1) Spin Multiplicity:
3
Cl1 O4 1- [Component No. 2]
Component(2) Charge:
-1
Component(2) Spin Multiplicity:
1
Generate Files Only
Confirm and Pass to MrPIXEL Console
Close SetupPixel

iv.

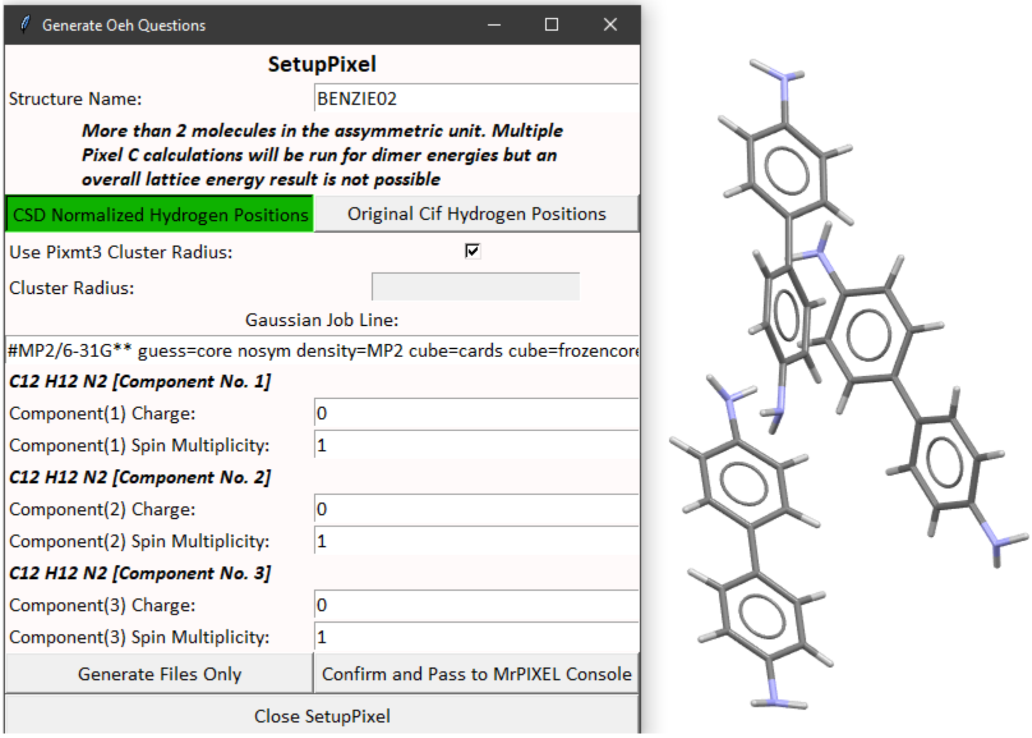

Figure S1: Example menus of SetupPixel and MRPixel. (i). SetupPIXEL for GLYCIN16 structure, (ii) symmetry lowering for ethylene, (iii) a two-component system where the cation is a transition metal complex, and (iv) a system for which Z' = 3.

Visualisation of Results with ProcessPixel

ProcessPixel is a program for visualising the results of Pixel calculations by means of energy vector plots (Bond, 2014, Shishkin *et al.*, 2012).

ProcessPixel expects a molecular centroid to correspond to a centre of gravity. However, by default we have used the geometric centre of the atomic coordinates for compatibility with the way the Mercury calculates centroids. These alternatives are selected in the file pixpar.par which is in the directory \MrPixel\Pixel\batch. Line 4 of this file reads

0.0 1

where the entry '1' defines centroids to be calculated as centries of coordinates. Should calculation of centres of gravity be required this line should be changed to

0.0 0

Full details are available in the Pixel manual.

ProcessPixel requires the .oeh and .mlc files produced by a Pixel calculation, and it can be invoked by issuing the command

ProcessPixel

at the command prompt in the directory containing the .oeh and .mlc files. The results of the calculation can then be processed as described in Bond (2014). For example, the energy vector plot for ETHLEN10 is shown in Fig. S2.

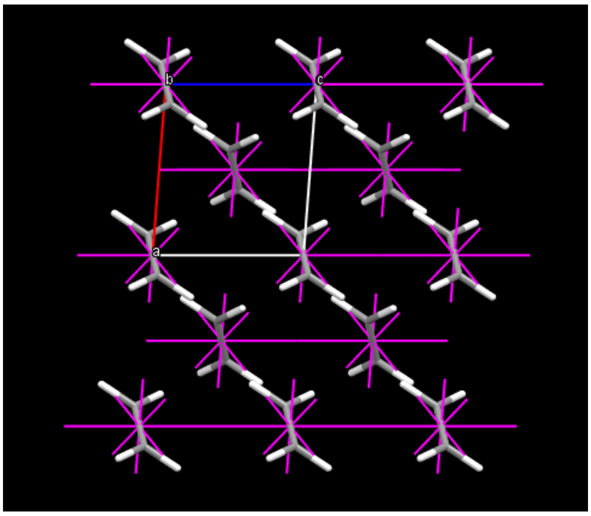

Figure S2: Enery vector plot for the crystal structure of ethylene (ETHLEN10). The view is along **b** with the *c*-axis horizontal. The strongest interactions are formed in lattice repeats along **c** (see Table 2 in the main text).

References:
Bond, A. (2014). *J. Appl. Cryst.* **47**, 1777-1780.
Shishkin, O. V., Dyakonenko, V. V. & Maleev, A. V. (2012). *CrystEngComm* **14**, 1795-1804.
